# Forecasting National and Regional Youth Unemployment in Spain Using Google Trends

**DOI:** 10.1007/s11205-022-02984-9

**Published:** 2022-08-19

**Authors:** Mihaela Simionescu, Javier Cifuentes-Faura

**Affiliations:** 1 Institute for Economic Forecasting of the Romanian Academy, Bucharest, Romania; 2 University of Murcia, Murcia, Spain

**Keywords:** Google trends, Youth unemployment, Pandemic, Panel data models

## Abstract

In Spain, the youth unemployment rate is one of the highest in the European Union. With the pandemic caused by Covid-19, young people face high unemployment rates and are more vulnerable to a decrease in labour demand. This paper analyses and predicts youth unemployment using Google Trends indices in Spain for the period between the first quarter of 2004 and the second quarter of 2021, being the first work to carry out this study for Spain and the first to use the regional approach for the country. Vector autoregressive Bayesian models and vector error correction models have been used for national data, and Bayesian panel data models and fixed effects model for regional data. The results confirm that forecasts based on Google Trends data are more accurate in predicting the youth unemployment rate.

## Introduction

Unemployment is a crucial issue for Spain. The recent economic crisis and the Covid-19 pandemic have deepened this social tension at national and regional level. High levels of youth unemployment in Spain are the main problem for this age group, which this country needs to alleviate through national policies. This country has registered higher values for youth unemployment rate compared to European average in the last decade. These high unemployment rates in Spain are explained not only by economic causes (eg., economic crises), but also by structural changes. Structural changes refer to the segmentation of the labor market, poor performance in education, the reduced capacity of the public sector to provide jobs for young people.

The Eurofound survey conducted in April-June 2020 to assess the impact of Covid-19 epidemic on young people in the EU revealed significant declines in well-being and the fact that young people are the category most affected by job losses. NEET ("Not in Education, Employment, or Training") young people were among the most affected people by the Great Recession from 2008 to 2013, and the effects of epidemic on them are expected to be even stronger. The explanations could be related to the fact that these young people tend to work more in sectors that have reduced their activity during the pandemic, have temporary contracts or work in precarious working conditions. Therefore, they are more susceptible to dismissal or reduced working time, which prevents them from entering the labor market or puts them at risk of long-term unemployment.

The consequences of youth unemployment are related to lower living standards (Mousteri et al., 2020), financial difficulties and psychological stress (Michaelides et al., 2019), lower wages after employment (Helbling et al., 2019), lower probability labor market insertion (Abebe & Hyggen, 2019) and greater propensity to engage in fraudulent activities (Dvouletý et al., 2019). In the long-run, the consequences of lack of a job among young people are related to reduced income for life, increased likelihood of unemployment in the coming periods, but also the chances of employment only for a short period, which can lead to poor health, low standard of living and a decrease in satisfaction due to professional achievements (Bell & Blanchflower, 2011). Compared to youth unemployment in the 1980s, current youth unemployment is characterized by a long-term trend of rising unemployment among certain categories of young people (O’Reilly et al., 2015). For example, young people whose parents lost their jobs in previous economic crises are more affected by unemployment.

More measures are needed to solve these problems: promoting lifelong learning, adapting the education system to the requirements of the current labor market, ensuring the transition from school to work and from one job to another, ensuring non-discriminatory access to places available work without discrimination. As youth unemployment in Spain is persistent, the most appropriate recommendations are active labor market policies (eg., better education schemes and institutions to ensure the transition from one form of education to the workplace) that prevent the transformation of the short-term unemployment in a structural (long-term) unemployment (Caporale & Gil-Alana, 2014). Spain receives support from the European Union (EU) to achieve a youth employment rate of 75%, with the share being calculated in the total working age population (20–64 years). The aim of better integration of young people on the labor market is to ensure economic growth and improve living standards.

Given the negative consequences of youth unemployment and the necessary policies to overcome this issue, accurate forecasts for this indicator are required. Predictions based on national statistics are realeased late and with intermediate and inaccurate values that are subject to revisions. In this context, real-time and accurate forecasts are required to propose the best economic and social policies in the struggle to support young people without job. A solution to this issue is given by the utilization of Internet data in predicting youth unemployment rate in Spain. Previous studies have focused on the prediction of national youth unemployment in France (Fondeur & Karamé, 2013) and in Italy (Naccarato et al., 2018) using Google Trends (GT). Starting from these previous achievements from literature, our study proposes few novel directions of research. First, the youth unemployment is analyzed and predicted using Google Trends Indexes (GTIs) in Spain where this issue is more acute compared to Italy and France, being the first study of this type for Spain. Second, none of the previous studies analyze the youth unemployment from a regional perspective, which is the subject of our research. Besides national youth unemployment rate, we analyzed this phenomenon using Google Trends also in Spanish regions in the same period. This regional approach could serve as robustness analysis to the national perspective. Third, if most of the previous studies employ monthly data, we focused on quarterly youth unemployment rate which are more recommended to be used in designing short-run and medium economic and social policies to enhance transition from school to work. Quarterly data to predict unemployment rate based on GT have been previously used by Naccarato et al. (2018) in forecasting Italian youth unemployment and by D’Amuri and Marcucci (2017) in forecasting US unemployment.

Specific methods are used to predict youth unemployment rate in Spain: Bayesian vector-autoregressive (BVAR) models and vector error correction (VEC) models for national data and Bayesian panel data models and fixed-effects model for regional data. The national and regional predictions of youth unemployment rate in Spain confirm that forecasts based on Google Trends were more accurate than those using only inflation rate on the horizon covering the beginning of the pandemic (2020:Q1-2021:Q2).

After this introduction, the next sections of the paper focus on literature review, data and methodological framework before the presentation of the results. The last part of the study provides few valuable conclusions.

## Literature Review

People tend to reveal information about what their main interests are when searching the Internet (Einav & Levin, 2014; Malhotra et al., 2004; Sherman-Morris et al., 2011; Yeh et al., 2018). From the data obtained through online search, social phenomena such as unemployment can be studied (Lasso & Snijders, 2016; D’Amuri & Marcucci, 2017; Jung et al., 2019; Simionescu, 2020).

The Google Trends index is frequently used to perform large search volumes (Jun et al., 2018) and obtain data sources through the Internet (Ahmed et al., 2017; Carrière-Swallow & Labbé, 2013; Chadwick et al., 2015; Dilmaghani, 2019). Google Trends provides temporal data to show the degree of search for a particular keyword in a specific period and a specific location (Nuti et al., 2014; Nagao et al., 2019, Fenga & Son-Turan, 2020). The Google Trends index takes values between 0 and 100. The higher the value, the higher the popularity of the search term in the selected period and region.

Google Trends has several advantages, as the search data is accessible in real time, and the daily nature of the data allows tracking a more detailed pattern of the responses given both in the period before and after. In contrast, most data that focus on an objective outcome, such as unemployment, tend to be available with a delay and are often subject to substantial revisions (Borup & Schütte, 2020; Eichenauer et al., 2020). Although Google Trends also has some limitation, as it focuses only on an aggregated picture of micro data behaviour and there is little information on the methodology (Simionescu, 2020).

The youth population has been one of the population groups most affected by the Covid-19 pandemic as they have a higher economic and social vulnerability with a lower labour participation rate than it already was (International Labor Organization, 2020), increasing youth unemployment (Blustein et al., 2020; Lambovska et al., 2021).

In recent years, initiatives have been launched to reduce youth unemployment. In 2013, a Youth Guarantee was established by the European Council to reduce the high levels of youth unemployment. This guarantee targets young people under 25 years of age called NEETs and they are expected to receive a job offer, an internship or training period or continuing education within four months of leaving school or becoming unemployed (Cabasés Piqué et al., 2016; Escudero & Mourelo, 2015; Tosun et al., 2019). Other policy initiatives on education and employment for young people in Europe were also launched such as Youth on the Move (European Commission, 2010) which is part of the Europe 2020 strategy for sustainable growth; and the Youth Opportunities Initiative and Youth Employment Support package. By 2019, more than 20 million young people have joined the Youth Guarantee scheme and of these, each year 3.5 million took up an offer of employment, further education, a traineeship or an apprenticeship (European Commission, 2018). Despite these data, the coverage of the Youth Guarantee has been low, reaching an average of 42% of the total potential beneficiary population (NEET).

The European Commission has recently launched a new initiative to strengthen the Youth Guarantee to eliminate the dynamics of exclusion and social vulnerability faced by young people due to the Covid-19 pandemic (European Commission, 2020).

In Spain, unemployment is one of the biggest problems today, especially among young people (De Luca et al., 2020; Rodríguez-Modroño, 2019; Strecker et al., 2021). Youth unemployment (under 25) stood at 38.38% in Spain at the end of the second quarter of 2021 according to data from the National Institute of Statistics, making it the country with the highest youth unemployment in the European Union at that date.

Most studies in the literature have tried to predict global unemployment rates in different countries, but very few focus on youth unemployment, even though it is one of the main problems today. Moreover, the vast majority of studies are conducted at the national level and there are very few at the regional level. There is some work carried out to predict the unemployment rate at the global level in Spain, but there is no study of youth unemployment, nor any carried out at the regional level.

González-Fernández and González-Velasco (2018) used the Google Trends Index to predict the unemployment rate in Spain employing an AR model and using unemployment as a keyword. Mulero and Garcia-Hiernaux (2021) also predicted unemployment in Spain using the SARIMA model, Principal Component Analysis and Forward Stepwise Selection. For the use of keywords, they used queries related to leading job search applications (e.g., InfoJobs, LinkedIn); searches related to Spanish unemployment centres (e.g., Employment office, SEPE); queries related to standard job searching terms (e.g., Job offers, How to Find a Job, etc.) or searches related to those companies that generate most employment in Spain (e.g., work in Inditex). Vicente et al. (2015) used the keywords oferta de trabajo, oferta de empleo to predict unemployment using Google Trends. All the papers highlight the importance of using Google Trends to predict unemployment.

Among the papers that attempt to predict youth unemployment using Google Trends is that of Naccarato et al. (2018), who predicted the youth unemployment rate for Italy at the national level using the keyword offerte di lavoro (job offer), using an ARIMA model and the VAR model. Fondeur and Karamé (2013) used the term emploi, which means job but also employment, to predict unemployment in France. They used a univariate model, a bivariate model and a Fourier decomposition reference model.

Barreira et al. (2013) argue that predicting youth unemployment rather than general unemployment is likely to be better predicted because young people use the Internet more than adults.

Our study uses both national and regional approaches. In fact, it is the first study to use the regional approach for Spain, and the first to do so for youth unemployment in Spain using Google Trends. This approach based on national and regional forecasts is important to make comparisons between unemployment tendencies at national and regional level. High predicted values of unemployment in Spanish regions might be reflected quickly or slower in the evolution at national level. The generalized increase in the predicted values of unemployment at regional level might show earlier that this challenge is a national issue that requires policy priority.

## Data and Methodology

This paper uses Google Trends to collect data about searching on Google for specific keywords related to unemployment. GTI takes the value zero for those queries with low search volume. The repeated searches from the same machine in a very short period are not taken into account, while the filtration of queries with apostrophes and special characters is made.

The values for GTIs were retrieved four various keywords (desempleo, InfoJobs, ofertas de empleo, ofertas de trabajo) on September 1st, 2021. The period covers the first quarter of 2004 up to the second quarter of 2021, when search terms for "All categories" are considered instead of topics and the geographic zone is represented by Spain. The individual searches were considered instead of comparisons. Quotes or strings that include " + " were not taken into consideration in the keywords.

This research is based on quarterly data at national level and regional level for Spain (2004:Q1-2021:Q2) for the same variables:

- Youth unemployment rate (seasonally adjusted): unemployment associated to young people under 25 years;
- Inflation rate (seasonally adjusted): it shows changes in prices being computed starting from consumer price index;
- GTIs for desempleo (unemployment);
- GTIs for InfoJobs;
- GTIs for ofertas de empleo (job vacancies);
- GTIs for ofertas de trabajo (job offers).

The 17 autonomous regions in Spain are considered in this analysis: Andalusia, Catalonia, Community of Madrid, Valencian Community, Galicia, Castile and León, Basque Country, Castilla-La Mancha, Canary Islands, Region of Murcia, Aragon, Extremadura, Balearic Islands, Asturias, Navarre, Cantabria, and La Rioja.

The use of inflation as exogenous variable to explain unemployment is justified by the Phillips curve. Usually, inflation rate values are released faster than unemployment and this could serve as timely indicator to predict unemployment. Nine historical views on Phillips curve are presented by Forder (2021), but the general economic theory considers that low unemployment might be maintained with the cost of high inflation. More indicators could be included in the models, but only inflation rate was selected because of limited availability of data for Spanish regions.

According to Table 1, a large range is observed for youth unemployment rate, from a minimum of 16.9% in the third quarter of 2006 to a maximum value of 56.9% in the first quarter of 2013 because of the economic recession. Before the economic crisis temporary contracts were concluded for half of the young people, but after the economic crisis start, these vulnerable workers were fired which doubled the youth unemployment rate compared to pre-crisis period (García, 2011). Inflation rose until 8% in the third quarter of 2009 at the beginning of the world economic crisis with a deep deflation in the third quarter of 2010. Lower values for GTIs in case of desempleo were observed compared to other keywords.

**Table 1:** Descriptive statistics for national data (2004:Q1-2021:Q2)

Variable	Mean	Maximum	Minimum	Standard deviation
Youth unemployment rate (%)	36.81765	56.9	16.9	12.52428
Inflation rate (%)	1.501471	8	− 7.2	1.933483
GTI for desempleo	28.75	76	15	9.346362
GTI for InfoJobs	55.11765	100	14	24.06058
GTI for ofertas de empleo	51.23529	99	29	13.61531
GTI for ofertas de trabajo	43.29412	100	19	15.23258

Source: own calculations in EViews

According to Table 2, there is a large range for youth unemployment rate, from a minimum of 7.29% in Balears Illes in the third quarter of 2007 to a maximum value of 69.69% in Canarias in the last quarter of 2012 because of the deep economic recession. The maximum inflation was registered in Cataluña in the third quarter of 2009, while the highest deflation was observed also in Cataluña in the third quarter of 2010. GTIs for all keywords ranged from 0 to 100.

**Table 2:** Descriptive statistics for regional data (2004:Q1-2021:Q2)

Variable	Mean	Maximum	Minimum	Standard deviation
Youth unemployment rate	35.26733	69.69	7.29	13.41738
Inflation rate	1.468882	10.3	− 8.81	1.908226
GTI for desempleo	15.09748	100	0	16.89916
GTI for InfoJobs	33.41261	100	0	24.50414
GTI for ofertas de empleo	19.53866	100	0	18.16404
GTI for ofertas de trabajo	18.57227	100	0	18.52145

Source: own calculations in Stata 17

The models at national and regional level are constructed for the period 2004:Q1-2019:Q4 and one-step-ahead forecasts are made on the horizon 2020:Q1-2021:Q2. Two types of models are built: models explining youth unemployment rate using only inflation, a macroeconomic indicator and models based only on GTIs for various keywords (desempleo, InfoJobs, ofertas de empleo, ofertas de trabajo).

Two types of models are considered at each level for robustness check. The models at national level are based on time series and consist in the following types: vector error correction (VEC) models and Bayesian vector autoregressive (BVAR) models. The models at regional level are based on panel data and refer to Bayesian panel data models and fixed-effects models.

The selection of Bayesian models as forecasting method compared to a frequentist approach based on VEC is justified by the fact that uncertainty is reduced by including prior information. Ma and Pigné (2019) recommend the use of dynamic Bayesian models for short-run forecasts like in our case. Moreover, other studies confirmed the superiority of Bayesian predictions. For example, Belloni (2017) showed that Bayesian VAR models outperformed VAR/VEC models when various macroeconomic indicators are predicted. Dynamic Bayesian panel data models are recommended by Liu et al. (2020) for predictions made under stressed macroeconomic conditions.

The vector error correction (VEC) model is built for two variables: youth unemployment rate (yu) and each of the other variables (inflation rate and GTIs). If the other variable is denoted by x, the form of the VEC model of order p (VEC(p)) is:

1 $$\begin{gathered} \Delta yu_{t} = \beta_{10} + \beta_{11} \Delta yu_{t - 1} + \cdots + \beta_{1p} \Delta yu_{t - p} + \gamma_{11} \Delta x_{t - 1} \hfill \\ \quad \quad \quad\;\, + \cdots + \gamma_{1p} \Delta x_{t - p} - \delta_{1} \left( {yu_{t - 1} - \alpha_{0} - \alpha_{1} x_{t - 1} } \right) + u_{1t} \hfill \\ \end{gathered}$$

2 $$\begin{gathered} \Delta x_{t} = \beta_{20} + \beta_{21} \Delta yu_{t - 1} + \cdots + \beta_{2p} \Delta yu_{t - p} + \gamma_{21} \Delta x_{t - 1} \hfill \\ \quad \quad \;\;\, + \cdots + \gamma_{2p} \Delta x_{t - p} - \delta_{2} \left( {yu_{t - 1} - \alpha_{0} - \alpha_{1} x_{t - 1} } \right) + u_{2t} \hfill \\ \end{gathered}$$

The long term cointegrating relationship between the two variables is given by ${yu}_{t}={\alpha }_{0}+{\alpha }_{1}{x}_{t}$.

${\delta }_{1},{\delta }_{2}$—error-correction coefficients measuring how the variables yu and x react to deviations from long term equilibrium.

Let us start from Eq. (3) that is equivalent with (4) to introduce Bayesian model with n variables and p lagged values:

3 $$y_{t}^{\prime } = \alpha + \mathop \sum \limits_{p = 1}^{L} y_{t - p}^{\prime } b_{i} + Dz_{t} + \varepsilon_{t}^{\prime }$$

4 $${\text{y}}_{{\text{t}}} = {\text{X}}_{{\text{t}}} \beta + \varepsilon_{t}$$

$\left( {\alpha^{\prime},b_{1}^{\prime } , \ldots ,b_{L}^{\prime } } \right) = B^{\prime}$-parameters of VAR model

${\mathrm{y}}_{\mathrm{t}}$ (column vector of dimension n including endogenous variables)

$${\text{X}}_{{\text{t}}} = \left( {{\text{I}}_{{\text{n}}} \otimes {\text{W}}_{{{\text{t}} - 1}} } \right)$$ (n × nk matrix)

$${\mathrm{W}}_{\mathrm{t}-1}=({\mathrm{y}}_{\mathrm{t}-1}^{\mathrm{^{\prime}}},{y}_{t-2}^{\mathrm{^{\prime}}},\dots ,{y}_{t-p}^{\mathrm{^{\prime}}},{z}_{t}^{\mathrm{^{\prime}}})$$ (k × 1).

${z}_{t}$- vector of exogenous variables ( d ×  1).

$\beta =vec({b}_{1},{b}_{2},\dots ,{b}_{p},D)$ (nk × 1) coefficients of BVAR model.

D- parameters matrix (n ×  d).

${\upvarepsilon }_{\mathrm{t}},{\varepsilon }_{t}^{^{\prime}}$- errors (iid, normal distribution).

$\Sigma$- covariance matrix of errors

$${\varepsilon }_{t}^{^{\prime}}\to N(0,\Sigma )$$

$\Sigma$ and B are the unknown parameters.

The likelihood function of the coefficients in Eq. (5) is combined with joint prior distribution of these parameters in Eq. (6), where ${ \ltimes }$ means proportional to:

5 $$L(y|\beta ,{\text{~}}\Sigma ){ \ltimes }\left| \Sigma \right|^{{ - \frac{{\text{T}}}{2}}} \left\{ { - \frac{1}{2}\mathop \sum \limits_{{\text{t}}} \left( {{\text{y}}_{{\text{t}}} - {\text{X}}_{{\text{t}}} \beta } \right)^{\prime } \Sigma ^{{ - 1}} \left( {{\text{y}}_{{\text{t}}} - {\text{X}}_{{\text{t}}} \beta } \right)} \right\}$$

6 $$p\left( {\beta ,\Sigma |{\text{y}}} \right) = \frac{{{\text{p}}\left( {\beta ,\Sigma } \right){\text{L}}({\text{y}}|\beta ,\Sigma )}}{{{\text{p}}\left( {\text{y}} \right)}}{ \ltimes }{\text{p}}\left( {\beta ,\Sigma } \right){\text{L}}({\text{y}}|\beta ,\Sigma )$$

In this case, we consider a multivariate normal prior for B and independent inverse Wishart prior for $\Sigma$. Under this type of prior distribution, the posterior distribution is Normal-Wishart (Karagöz & Keskin, 2016).

The same Bayesian principle is applied for Bayesian panel data models. The fixed-effects regression model for two variables yu and x (i-index for region, t-index for quarter) is given by:

7 $$yu_{it} = \theta_{i} + \vartheta x_{it} + v_{it}$$

${\theta }_{i}$-region-specific intercepts capturing heterogeneities across regions; $\vartheta$-parameter; ${v}_{it}$-error term.

All these models are used to make predictions for youth unemployment rate in Spain (national level and regional level). The accuracy of these forecasts is assessed using Root mean squared error (RMSE) and U2 Theil’s coefficient. The forecast error at time *t* (${e}_{t}$) is based on the comparison between the registered value for that variable at time *t* (${y}_{t}$) and the corresponding forecast at the same time (${\widehat{y}}_{t}$): ${e}_{t}={y}_{t}-{\widehat{y}}_{t}$. If h is the horizon length and n is the moment corresponding to the last value in the time series, the RMSE is computed as:

8 $$RMSE = \sqrt {\frac{1}{h}\mathop \sum \limits_{t = n + 1}^{n + h} \left( {y_{t} - \hat{y}_{t} } \right)^{2} }$$

The U1 Theil’s coefficient is employed to compare forecast accuracy and the U2 Theil’s coefficient is used to make comparisons with naïve forecasts:

9 $$U_{1} = \frac{{\sqrt {\mathop \sum \nolimits_{t = n + 1}^{n + h} \left( {y_{t} - \hat{y}_{t} } \right)^{2} } }}{{\sqrt {\mathop \sum \nolimits_{t = n + 1}^{n + h} y_{t}^{2} } + \sqrt {\mathop \sum \nolimits_{t = n + 1}^{n + h} \hat{y}_{t}^{2} } }}$$

10 $$U_{2} = \sqrt {\frac{{\mathop \sum \nolimits_{t = n + 1}^{n + h - 1} \left( {\frac{{\hat{y}_{t + 1} - y_{t + 1} }}{{y_{t} }}} \right)^{2} }}{{\mathop \sum \nolimits_{t = n + 1}^{n + h - 1} \left( {\frac{{y_{t + 1} - y_{t} }}{{y_{t} }}} \right)^{2} }}}$$

A value close to 0 for U1 Theil’s coefficient indicates high forecast accuracy. A value less than 1 for U2 Theil’s coefficient indicates that the analyzed forecasts are better than naïve forecasts that uses random walk.

An accuracy test should also be applied to compare predictions performance. In this case, Diebold-Mariano (DM) test is used under the null hypothesis of equal accuracy. This test is based on a regression model for the difference in squared errors corresponding to predictions that are compared.

## Results

The presentation of the results is made separately for national and regional level and comparisons are made in the end.

### Results- National Approach

First, the stationary character of the time series is checked using ADF test. The results are presented in Table 3, where critical values at 5% significance level are shown in brackets.

**Table 3:** Results of unit root tests for data in level and in the first difference

Variable	ADF stat. (trend)	ADF stat. (trend and intercept)
*Time series in level for*		
Youth unemployment rate	− 2.192237 (− 2.908420)	− 2.753346 (− 3.482763)
Inflation rate	− 2.342678 (− 2.906744)	− 3.055347 (− 3.482763)
GTI for desempleo	− 4.346974 (− 2.905519)	− 1.581136 (0.7894)
GTI for InfoJobs	− 0.902938 (0.7811)	− 3.113573 (0.1123)
GTI for ofertas de empleo	− 2.878602 (− 2.908420)	− 3.205998 (− 3.482763)
GTI for ofertas de trabajo	− 3.407394 (− 3.482763)	− 1.912529 (− 2.908420)
*Time series in the first difference for*		
Youth unemployment rate	− 2.992490 (− 2.908420)	− 4.384894 (− 3.482763)
Inflation rate	− 5.653557 (− 2.905519)	− 6.703936 (− 3.478305)
GTI for desempleo	− 3.797137 (− 2.908420)	− 4.999628 (− 3.482763)
GTI for InfoJobs	− 2.912328 (− 2.908420)	− 4.534283 (− 3.482763)
GTI for ofertas de empleo	− 4.700781 (− 3.482763)	− 2.912529 (− 2.908420)
GTI for ofertas de trabajo	− 4.584405 (− 2.910860)	− 4.532009 (− 3.486509)

Source: own calculations in EViews

According to results in Table 3, the time series for all variables are integrated of order 1 at 5% significance level. Therfore, a possible relationship of cointegration between these series is checked. The Johansen test is applied and the number of cointegation relationships is presented in Table 4. Two versions of the Johansen test are considered: the test based on trace and the one based on maximum eigenvalue (Bilgili et al., 2017). In both cases, the null hypothesis states that no cointegration relationship is detected.

**Table 4:** The results of Johansen test

Data trend	None	None	Linear	Linear	Quadratic
Test type	No intercept	Intercept	Intercept	Intercept	Intercept
	No trend	No trend	No trend	Trend	Trend
Trace	3	3	3	4	4
Max-Eig	3	3	3	3	3

Source: own calculations in EViews

More relationships of cointegration are identified by Johansen test. Excepting linear and quadratic equations with trend and intercept for the test based on trace, the results indicate the existence of 3 cointegration relationships. In this case, we consider the existence of 3 cointegration relationships for linear model with intercept. Therefore, some VEC models are built on data in level. These models are built for the period 2004:Q1-2019:Q4 and forecasts are made on the horizon 2020:Q1-2021:Q2.

Three valid VEC models were built based on youth unemployment rate and the following variables: inflation rate, GTI for desempleo and GTI for InfoJobs. Only the equations explaining youth unemployment rate are presented below since these equations are used to predict this indicator. The optimal lags of the VEC models were selected using Akaike information criterion.

$$\begin{aligned} {\text{VEC1}}: & \Delta yu_{t} = 0.006832 \cdot \left( {yu_{t - 1} + 18.38472 \cdot inflation_{t - 1} - 65.17173} \right) \\ & \quad\quad\quad\;+ 0.368913 \cdot \Delta yu_{t - 1} - 0.119542 \cdot \Delta inflation_{t - 1} + 0.163187 \\ \end{aligned}$$

$$\begin{aligned} VEC2: & \Delta yu_{t} = - 0.041326 \cdot \left( {yu_{t - 1} - 2.355764 \cdot GTI_{t - 1}^{desempleo} + 30.67762} \right) + 0.339741 \cdot \Delta yu_{t - 1} \\ & \quad\quad\quad\;- 0.194015 \cdot \Delta yu_{t - 2} + 0.000767 \cdot \Delta GTI_{t - 1}^{desempleo} - 0.034708 \cdot \Delta GTI_{t - 2}^{desempleo} + 0.262171 \\ \end{aligned}$$

$$\begin{aligned} VEC3: & \Delta yu_{t} = - 0.029849 \cdot \left( {yu_{t - 1} - 0.092042 \cdot GTI_{t - 1}^{InfoJobs} - 31.83306} \right) \\ & \quad\quad\quad\;\,+ 0.369678 \cdot \Delta yu_{t - 1} - 0.018616 \cdot \Delta GTI_{t - 1}^{InfoJobs} + 0.161324 \\ \end{aligned}$$

The errors are homoskedastic, independent up to lag 10 and normally distributed as Table 5 shows. According to equations above, there is a significant long-run relationship between youth unemployment rate and GTI for desempleo and between youth unemployment rate and GTI for InfoJobs. A long-run connection between inflation and youth unemployment rate is not observed. These VEC models are used to make forecasts for youth unemployment rate on the horizon 2020:Q1-2021:Q2.

**Table 5:** Diagnostic tests for VEC models used to explain the relationship between youth unemployment rate in Spain, inflation rate and GTIs for desempleo and InfoJobs

Statistics of tests	VEC1	VEC2	VEC3
Chi-square stat. (White test with *p* value in brackets)	15.90636 (0.5991)	17.53755 (0.4865)	16.76684 (0.5033)
Jarque–Bera stat. (*p* value in brackets)	3.871915 (0.1443)	4.354857 (0.1133)	0.455186 (0.7964)

Source: own calculations in EViews

For robustness, more BVAR(4) models for youth unemployment rate and other variables (variation in inflation rate and GTIs (keywords: desempleo, InfoJobs, oe (ofertas de empleo), ot (ofertas de trabajo)) were built on data in the first difference to ensure stationarity. Table 6 presents only the equations explaining variation in youth unemployment rate that are used to predict this indicator. The lag equals 4 since the most of the information criteria (Akaike, Schwarz, Hannan-Quinn information criteria) indicate this value. MCSE is the Monte Carlo Standard Error that estimates the inaccuracy associated to Monte Carlo samples.

**Table 6:** BVAR(4) models for variation in youth unemployment rate (yu) and other variables

Variable	Mean	Standard deviation	MCSE	Median
*BVAR 1*				
$\Delta {yu}_{t-1}$	0.699	0.091	0.0009	0.700
$\Delta {yu}_{t-2}$	− 0.047	0.058	0.0006	− 0.048
$\Delta {yu}_{t-3}$	0.019	0.041	0.0004	0.019
$\Delta {yu}_{t-4}$	0.019	0.031	0.0003	0.019
$\Delta {inflation}_{t-1}$	− 0.029	0.0844	0.0008	− 0.029
$\Delta {inflation}_{t-2}$	− 0.0006	0.055	0.0006	− 0.001
$\Delta {inflation}_{t-3}$	0.007	0.038	0.0004	0.007
$\Delta {inflation}_{t-4}$	− 0.002	0.029	0.0003	− 0.002
Constant	0.089	0.273	0.003	0.091
*BVAR 2*				
$\Delta {yu}_{t-1}$	0.698	0.090	0.0009	0.698
$\Delta {yu}_{t-2}$	− 0.045	0.057	0.0005	− 0.046
$\Delta {yu}_{t-3}$	0.020	0.039	0.0003	0.019
$\Delta {yu}_{t-4}$	0.019	0.030	0.0003	0.019
$\Delta {GTI}_{t-1}^{desempleo}$	0.030	0.021	0.0002	0.030
$\Delta {GTI}_{t-2}^{desempleo}$	− 0.006	0.014	0.0001	− 0.006
$\Delta {GTI}_{t-3}^{desempleo}$	0.002	0.009	0.00009	0.002
$\Delta {GTI}_{t-4}^{desempleo}$	− 0.0004	0.007	0.00007	− 0.0005
Constant	0.805	0.269	0.0027	0.079
*BVAR 3*				
$\Delta {yu}_{t-1}$	0.715	0.087	0.0008	0.716
$\Delta {yu}_{t-2}$	− 0.036	0.055	0.0005	− 0.036
$\Delta {yu}_{t-3}$	0.015	0.038	0.0003	0.016
$\Delta {yu}_{t-4}$	0.014	0.029	0.0002	0.014
$\Delta {GTI}_{t-1}^{InfoJobs}$	− 0.014	0.021	0.0002	− 0.014
$\Delta {GTI}_{t-2}^{InfoJobs}$	0.010	0.017	0.0002	0.009
$\Delta {GTI}_{t-3}^{InfoJobs}$	0.021	0.012	0.0001	0.021
$\Delta {GTI}_{t-4}^{InfoJobs}$	− 0.012	0.010	0.0001	− 0.012
Constant	0.069	0.255	0.003	0.066
*BVAR 4*				
$\Delta {yu}_{t-1}$	0.716	0.088	0.0008	0.716
$\Delta {yu}_{t-2}$	− 0.039	0.055	0.0006	− 0.040
$\Delta {yu}_{t-3}$	0.019	0.039	0.0004	0.019
$\Delta {yu}_{t-4}$	0.015	0.029	0.0003	0.015
$\Delta {GTI}_{t-1}^{oe}$	− 0.011	0.018	0.0002	− 0.012
$\Delta {GTI}_{t-2}^{oe}$	0.005	0.014	0.0001	0.006
$\Delta {GTI}_{t-3}^{oe}$	0.017	0.011	0.0001	0.018
$\Delta {GTI}_{t-4}^{oe}$	− 0.012	0.008	0.00008	− 0.012
Constant	0.095	0.262	0.002	0.098
*BVAR 5*				
$\Delta {yu}_{t-1}$	0.713	0.088	0.0008	0.713
$\Delta {yu}_{t-2}$	− 0.038	0.055	0.0005	− 0.038
$\Delta {yu}_{t-3}$	0.014	0.039	0.0004	0.015
$\Delta {yu}_{t-4}$	0.015	0.029	0.0003	0.015
$\Delta {GTI}_{t-1}^{ot}$	− 0.015	0.020	0.0002	− 0.016
$\Delta {GTI}_{t-2}^{ot}$	0.007	0.015	0.0002	0.007
$\Delta {GTI}_{t-3}^{ot}$	0.016	0.011	0.0002	0.016
$\Delta {GTI}_{t-4}^{ot}$	− 0.011	0.008	0.00008	− 0.011
Constant	0.064	0.258	0.002	0.062

Source: own calculations in Stata 17

A sample of MCMC values is associated to a Bayesian forecast for a certain quarter instead of a single value. These multiple values are aggregated by computing mean and median that represent single statistics. Therefore, two types of Bayesian dynamic forecasts are built using each regression: prediction based on posterior mean and prediction based on posterior median. The one-step-ahead forecasts on short horizon cover the pandemic period (2020:Q1-2021:Q2). RMSE, U1 and U2 Theil’s coefficient are computed as accuracy measures for the forecasts based on the proposed models. The U2 Theil’s coefficient allow us to make comparisons with naive forecasts based on random walk, the values being computed in Table 7.

**Table 7:** The forecast accuracy for youth unemployment rate predictions in Spain (horizon: 2020:Q1-2021:Q2)

Accuracy measure	BVAR 1		BVAR 2		BVAR 3		BVAR 4		BVAR 5		VEC1	VEC2	VEC3
RMSE	0.561	1.588	0.355	0.350	0.478	0.471	0.558	0.569	0.557	0.528	0.722	0.634	0.645
U1 coef	0.677	0.898	0.492	0.455	0.527	0.533	0.653	0.657	0.634	0.602	0.794	0.756	0.787
U2 coef	1.256	0.968	0.811	0.821	0.801	0.807	0.951	0.933	0.676	0.714	0.894	0.778	0.802

Source: own calculations

According to RMSE and U1 Theil’s coefficient, the Bayesian dynamic forecasts based on BVAR 2 models provide the most accurate forecasts for youth unemployment rate on the horizon 2020:Q1-2021:Q2. This model is based on GTIs for keyword desempleo. The forecasts based on posterior median associated to BVAR 2 performed better than those based on posterior mean. According to Diebold-Mariano test, there is not a significant difference in accuracy between the two BVAR2 forecasts (DM stat. = 0.455, *p* value = 0.703).

All the predictions were more accurate than naive forecasts, excepting the Bayesian dynamic forecasts based on posterior mean of BVAR 1 that is not based on GT data. Bayesian dynamic forecasts were better than those based on VEC models in terms of accuracy. All in all, we can conclude that forecasts based on GT data for youth unemployment rate in Spain perform better than these using only an official statistic (inflation rate) that is usually released later.

### Results- regional approach

Preliminary tests are considered under the assumption of regions’ heterogeneity due to regional gaps in youth unemployment rate: test for cross-sectional dependence (CD) and panel unit root test to determine the order of integration. The CD test developed by Pesaran et al. (2008) is applied to check for contemporaneous correlations across regions (see Table 8).

**Table 8:** The results of CD test

Variable	CD test stat
Youth unemployment rate	86.61*
Inflation rate	95.52*
GTI for desempleo	13.66*
GTI for InfoJobs	44.51*
GTI for ofertas de empleo	10.82*
GTI for ofertas de trabajo	16.87*

Source: own calculations in Stata 17

*means *p* value less than 0.05

According to CD test results, contemporaneous correlations across regions are present for all variables. The cross-sectional dependence is explained by the common regulations for all regions according to Spanish labour market, common programs for vulnerable groups in the labour field and other similarities of economic and social nature.

Under heterogeneity and cross-sectional dependence with balanced panels (no missing data), Breitung test is applied to check for unit root. The results of this test are presented in Table 9.

**Table 9:** The results of Breitung test

Variable	Calculated statistics (data in level)	
	No lag	One lag
Youth unemployment rate	− 2.65098*	− 3.0680*
Inflation rate	− 13.6098*	− 8.7756*
GTI for desempleo	− 17.5053*	− 9.2521*
GTI for InfoJobs	− 8.2795*	− 2.7348*
GTI for ofertas de empleo	− 16.2290*	− 10.0073*
GTI for ofertas de trabajo	− 8.2352*	− 6.3264*

Source: own calculations in Stata 17

*means *p* value less than 0.05

According to Table 9, panel data are stationary in level for all variables at 5% significance level. Threfore, data in level are used to construct Bayesian panel data models (BP1, BP2, BP3, BP4, BP5) as Table 10 indicates. The panel data models are constructed for the period 2004:Q1-2019:Q4 and forecasts are made on the horizon 2020:Q1-2021:Q2.

**Table 10:** The Bayesian panel data models to explain youth unemployment rate in Spanish regions

Variable	Mean	Standard deviation	MCSE	Median
BP1				
${inflation}_{t}$	− 2.281372	0.1781802	0.005146	− 2.28575
Constant	38.38001	1.448045	0.422338	38.27571
BP2				
${GTI}_{t}^{desempleo}$	0.154473	0.0247549	0.001241	0.154579
Constant	32.17279	1.354421	0.326271	32.10346
BP3				
${GTI}_{t}^{InfoJobs}$	0.0303191	0.0173742	0.000826	0.0304544
Constant	34.64249	1.53678	− .354563	34.06314
BP4				
${GTI}_{t}^{oe}$	− 0.0596001	0.0244423	0.00138	− 0.060077
Constant	35.97142	1.172696	0.264742	35.97814
BP5				
${GTI}_{t}^{ot}$	− 0.0765812	0.0218221	0.000856	− 0.0761495
Constant	36.88127	1.47002	0.404029	36.7005

Source: own calculations in Stata 17

The convergence of MCMC is checked. The graphs in Appendix 1 suggest no apparent trend, because the autocorrelation tends to decrease in time. The posterior distributions for all the regions are presented in Appendix 2 and suggest variation among regions’ youth unemployment. For example, when inflation is considered in the model, regions like Andalusia, Principado de Asturias and La Rioja present higher youth unemployment compared to the rest of the regions. This conclusion slightly changes when GTIs for various keywords are considered in the models. Canarias has higher youth unemployment rates when desempleo and ofertas de empleo are variables in the Bayesian panel data models.

Posterior predictive *p* values (known as PPPs) are computed to perform posterior predictive checks. These values show how often the computed statistics based on MCMC sample represent extreme values compared to other values. The PPPs associated to maximum and minimum values should not be close to 0 or 1 to avoid extreme values that induce poor fit to model. The PPPs for minimum (BP1: 0.32, BP2: 0.45, BP3: 0.37, BP4:0.42, BP5:0.43) and for maximum (BP1: 0.67, BP2: 0.72, BP3:0.77, BP4:0.79, BP5:0.81) are not very close to 0 or 1.

For robustness, fixed-effects models with robust standard errors are built (FE1, FE2, FE3, FE4, FE5), because they are better than random effects models according to Hausman test (see Table 11).

**Table 11:** The fixed-effects panel data models to explain youth unemployment rate in Spanish regions

Variable	Coefficients				
	FE1	FE2	FE3	FE4	FE5
Constant	38.61205*	32.79626*	34.13748*	36.42321*	36.7009*
${inflation}_{t}$	− 2.27705*	–	–	–	–
${GTI}_{t}^{desempleo}$	–	0.1636744*	–	–	–
${GTI}_{t}^{InfoJobs}$	–	–	0.033815**	–	–
${GTI}_{t}^{oe}$	–	–	–	− 0.0591587*	–
${GTI}_{t}^{ot}$	–	–	–	–	− 0.0771888*

Source: own calculations in Stata 17

*means *p* value less than 0.05, **means *p* value less than 0.1

The Bayesian forecasts are built using posterior mean and posterior median. Some forecast accuracy measures are computed for each region (see Table Table 12

**Table 12:** The forecast accuracy for youth unemployment rate predictions in Spanish regions (horizon: 2020:Q1-2021:Q2)

Region	RMSE										U2 less than 1 compared to the best prediction	U1 for the best prediction
	Forecasts based on posterior mean					Forecasts based on posterior median						
	BP1	BP2	BP3	BP4	BP5	BP1’	BP2’	BP3’	BP4’	BP5’		
Andalucía	37.39063	40.04583	**34.50484**	34.72779	36.09157	37.72918	41.21094	34.62794	35.19193	36.08366	Yes	0.796
Aragón	37.7622	34.72096	34.57085	34.81706	35.57205	38.09876	35.56832	34.70123	35.27986	35.55706		0.783
Principado de Asturias	37.90332	34.04304	34.36774	35.26751	36.25668	38.23965	34.85023	34.4757	35.72342	36.25103		0.887
Balears Illes	37.38323	33.34248	34.19513	35.59776	36.32005	37.72135	**34.10827**	34.28404	36.04864	36.31526	Yes	0.804
Canarias	36.82484	36.9969	34.58102	34.95795	36.23127	37.16645	37.97844	34.71253	35.41857	36.22527		0.799
Cantabria	37.61002	**32.67855**	34.26618	35.88804	34.30652	37.94749	33.14219	34.36293	36.33447	34.27431	Yes	0.543
Castilla y León	37.88697	36.14709	34.51498	35.0074	35.96457	38.22293	37.07866	34.63919	35.46728	35.95493		0.711
Castilla–La Mancha	37.87618	**33.44703**	34.37279	35.11734	35.96457	38.21188	34.21903	34.48131	35.57555	35.95493	Yes	0.649
Cataluña	37.87349	40.76599	34.54036	34.96787	34.91256	38.21007	41.971	34.66737	35.42834	34.88858		0.704
Comunitat Valenciana	37.59547	39.22098	34.51499	**34.13896**	34.84979	37.93284	40.3336	34.6392	34.61204	34.82497	Yes	0.655
Extremadura	37.25883	**34.14463**	34.2611	35.64776	36.54823	37.59775	34.95775	34.35729	36.09787	36.54655	Yes	0.675
Galicia	37.4251	35.77682	**34.44898**	35.20839	35.72521	37.76295	36.68628	34.5659	35.66518	35.71232	Yes	0.676
Comunidad de Madrid	37.9754	39.79464	34.59623	**33.84871**	34.78631	38.31201	40.94122	34.72942	34.32622	34.76061	Yes	0.658
Región de Murcia	37.5364	33.62614	34.35763	35.46789	35.96564	37.87436	34.40863	34.46448	35.92074	35.95603	Yes	0.665
Comunidad Foral de Navarra	37.82094	**32.87975**	34.41858	35.35754	35.90125	38.15703	33.61835	34.53216	35.81208	35.89074	Yes	0.556
País Vasco	37.20911	37.57452	34.66729	34.65988	35.74935	37.54826	38.59039	34.80831	35.125	35.73678		0.745
La Rioja	38.10986	32.87732	34.35257	35.42814	36.39598	38.44532	33.61569	34.45886	35.88159	36.39223		0.877
Region	FE1	FE2	FE3	FE4	FE5							
Andalucía	37.65804	37.22505	34.53111	34.72724	35.91908							0.859
Aragón	37.79945	**34.32834**	34.48294	35.01655	35.84895						Yes	0.697
Principado de Asturias	37.66041	**33.65683**	34.29253	35.39294	36.26723						Yes	0.756
Balears Illes	36.65748	35.09096	34.35138	35.34426	36.27773							0.811
Canarias	37.59838	34.93134	**34.45355**	35.33596	35.387						Yes	0.762
Cantabria	37.80911	34.59684	34.37898	35.50572	35.07224							0.754
Castilla y León	37.8735	34.81376	**34.44733**	35.06737	35.96457						Yes	0.578
Castilla–La Mancha	37.74306	37.32663	34.44484	35.03938	35.53715							0.698
Cataluña	37.88873	39.82998	**34.53106**	34.66151	34.88138						Yes	0.794
Comunitat Valenciana	37.38391	36.87039	34.40771	34.76261	35.55014							0.745
Extremadura	37.41532	34.98846	34.32468	35.44401	36.18607							0.678
Galicia	37.40829	37.74273	34.52519	34.68188	35.31439							0.766
Comunidad de Madrid	37.90482	36.93098	34.49154	34.51057	35.33181							0.794
Región de Murcia	37.82169	**33.2537**	34.39064	35.44442	35.94385						Yes	0.767
Comunidad Foral de Navarra	37.41167	35.2911	34.50844	35.01894	35.81487							0.802
País Vasco	37.60997	35.34624	**34.55594**	35.00907	36.0358						No	0.778
La Rioja	15.55829	**13.42211**	14.02438	14.46348	14.8586							0.223

Bold numbers indicate the lowest value of RMSE for the corresponding forecast of a region

Source: own calculations

The results in Table 12 confirm that forecasts based on Google Trends data are more accurate than those using only inflation rate in predicting youth unemployment rate. Excepting País Vasco, the most accurate forecasts are better than naïve predictions. According to U1 coefficient, the predictions for La Rioja based on fixed effect models are the most accurate, being followed by the Bayesian forecasts for Cantabria.

According to Simionescu (2020), besides accuracy measures, at least one accuracy test should be applied to confirm the results. Given the intensive computations, in this case, the most accurate Bayesian forecast based on posterior mean is compared with the most accurate prediction based on fixed effect models. The Diebold-Mariano (DM) test is applied, and the results are presented in Table 12.

The equal forecast accuracy is not rejected for Canarias, Castilla y León, Cataluña, Región de Murcia, País Vasco at 5% significance level. For the rest of the forecasts, excepting Aragón, Principado de Asturias, La Rioja, DM test indicated that forecasts based on Bayesian models were more accurate than those based on fixed effect models.

Given these results, more clusters could be identified as Table 13 shows. In nine Spanish regions, the most accurate forecasts were based on GTIs associated to the keyword desempleo (Balears Illes, Cantabria, Castilla–La Mancha, Extremadura, Comunidad Foral de Navarra, Aragón, Principado de Asturias, Región de Murcia, La Rioja). In six Spanish regions, the predictions based on GTIs for InfoJobs perfomered the best (Andalucía, Galicia, Canarias, Castilla y León, Cataluña, País Vasco). Only for Comunidad de Madrid and Comunidad de Valencia, the forecasts based on GTIs for ofertas de empleo were the most accurate. The Bayesian forecasts were the best predictions in the case of 9 regions, while for 8 regions fixed-effects model were the most accurate.

**Table 13:** Distribution of Spanish regions according to most accurate forecasts on the horizon 2020:Q1-2021:Q2

Bayesian forecasts based on posterior median and GTIs for desempleo	Bayesian forecasts based on posterior mean and GTIs for desempleo	Bayesian forecasts based on posterior mean and GTIs for InfoJobs	Bayesian forecasts based on posterior mean and GTIs for ofertas de empleo	Forecasts based fixed-effects model and GTIs for desempleo	Forecasts based fixed-effects model and GTIs for InfoJobs
Balears Illes	Cantabria	Andalucía	Comunidad de Madrid	Aragón	Canarias
	Castilla–La Mancha	Galicia	Comunidad de Valencia	Principado de Asturias	Castilla y León
	Extremadura			Región de Murcia	Cataluña
	Comunidad Foral de Navarra			La Rioja	País Vasco
One region	4 regions	2 regions	2 regions	4 regions	4 regions

Source: own synthesis

These results are in line with the results for national level where the Bayesian dynamic forecasts based on models using GTIs for desempleo provide the most accurate forecasts for youth unemployment rate on the horizon 2020:Q1-2021:Q2.

Previous studies for Spain confirm the superiority of unemployment forecasts based on GTIs for monthly data: González-Fernández and González-Velasco (2018) obtained better predictions for unemployment compared to those based on random walk in the period January 2004-November 2017. Google Trends also improved the unemployment rate forecasts based on SARIMA models in Spain in the period January 2004-September 2018 (Mulero & García-Hiernaux, 2021).

## Discussion and Conclusion

The Covid-19 pandemic has caused young people, more intensely than other age groups, to experience a rapid drop in employment, making them a more vulnerable population. In Spain, the youth unemployment rate is one of the highest in the European Union and is one of the country's major problems.

Given that people tend to reveal information when searching on the Internet, it is possible to analyse phenomena such as unemployment through online search. This article uses Google Trends to collect data on Google searches for specific keywords related to unemployment. Four keywords have been used in the search: *desempleo*, *InfoJobs*, *ofertas de empleo* and *ofertas de trabajo*. The period analysed is from the first quarter of 2004 to the second quarter of 2021. The quarterly youth unemployment rate has been used, which is more suitable for designing short and medium-term economic and social policies.

For the analysis, Bayesian vector-autoregressive models and vector error correction models have been used for national data, and Bayesian panel data models and fixed effects model for regional data.

In nine Spanish regions, the most accurate forecasts were based on GTIs associated to the keyword *desempleo*. The Bayesian forecasts were the best predictions in the case of 9 regions, while for 8 regions fixed-effects model were the most accurate, these results being in line with those obtained at the national level, where dynamic Bayesian forecasts based on models using GTIs for *desempleo* provide the most accurate forecasts for the youth unemployment rate. Our findings confirmed the results of other study that show the superiority of GT data in predicting quarterly unemployment (for example, the study of Naccarato et al. (2018) that forecasted the Italian youth unemployment and the paper of D’Amuri and Marcucci (2017) that used GT data to predict US unemployment).

Forecasts based on Google Trends data are more accurate in predicting the youth unemployment rate than those using only the inflation rate. These results have several policy implications. If higher unemployment is predicted in real time in some regions, policymakers could make quick decisions to alleviate this phenomeon. If more succesive quarters are characterized by high levels of regional unemployment and an ascending trend is observed at national level as well, this might be a hint for entrance in economic recession. If the econometric model based on Okun law is considered, unemployment rate forecasts based on GT could be used to predict GDP at national and regional level. If the GDP forecasts indicate economic decline for two succesive quarters, the economic crisis might be declared.

The knowlege of the economic situation in advance could help government to take earlier decisions to support the economy and alleviate the negative consequences of the economic crisis. In the short-run, incentives could be ensured to support business environment and also vulnerable population without job or those with low wages and low qualification that have more chances to get hired. In the long-run, the policymakers should support the creation of new jobs, including legislative facilities for start-ups. Favourable legislation that promotes a friendly business environment to attract foreign direct investment is required. However, it is necessary to assign priority to those FDI projects in green and renewable sectors. New jobs could be created in these sectors. According to World Economic Forum report, the demand for green talents is higher than the supply (Odiyo et al., 2022). Moreover, the transition to green economy is also required by the threat of climate changes. Green jobs could cover many types of sectors, including construction, sales, healthcare, and research and could be an important solution to high unemployment predicted by Google Trends. Investment in education is also necessary to help people developing green skills. Start-ups hiring people with green skills should also be encouraged. According to World Economic Forum (2020), Spain is among the countries with high economic disruption, but with high social resilience. If the economic crisis is predicted in time, the social resilience will help Spanish government to allieviate the expected negative effects.

In the case of a potential economic crisis predicted by Google Trends data, long-run policies should be implemented to reduce income inequality. Spain is placed among the most unequal countries in the EU (the fourth place, after Greece, Romania and Bulgaria) (Cabrera et al., 2021). The last world economic crisis has intensified the issue of income inequality in Spain and it is expected to enhance it as well in the next financial crisis. In this case, income inequality should be reduced by considering improvement of human capital to reach necessary skills for labour market, expansion of infrastructure, higher minimum salary.

Simultaneous analysis of national and regional unemployment forecasts based on GT could provide useful information to anticipate an economic crisis. A generalizated rise of unemployment in Spanish regions accompanied by a high national unemployment prediction represents an important indice for an economic crisis. Moreover, more searches for jobs using Internet are also threats that might help regional and national governance to react in time.

The financial crisis predicted by Google Trends in Spain using unemployment rate forecasts could even be avoided if some of the recommendations of Beker (2021) are followed: the loans should not be too much concentrated in a single sector or region, national bank should not be lender of last resort, changes in compensations schemes, better regulation of conflict of interests that might appear in the agencies that make credit rating evaluation. Loans should not be assigned to those Spanish regions with high predicted unemployment rates to avoid a financial crisis.

Besides the utility of GT data in predicting unemployment and an eventual economic crisis, this study still presents few limitations. From methodological point of view, only few types of models were used to make predictions. In the M competitions organized to select the best method in predicting economic indicators, a large number of models is used (Fildes, 2020). However, in a future study, more types of methods will be used, including machine learning methods. Another limitation of this study is the consideration of a short horizon for forecasts, but the forecasting methods could be applied for longer horizons. The results focus on unemployment in Spain, but for other countries one should check if these forecasting methods provide accurate predictions for unemployment. Moreover, other explanatory macroeconomic variables could be introduced in the models, but we resumed to inflation rate because of data availability at regional level. In a future study, more countries will be considered when unemployment is predicted using GT data and more explanatory variables will be introduced in the models.

## Appendix 1

Convergence of MCMC

## Appendix 2

Posterior distributions.

INFLATION

DESEMPLEO

INFOJOBS

OFERTAS DE EMPLEO

OFERTAS DE TRABAJO
